# Supplementary material for: Description, reliability and utility of a ground-reaction-force triggered protocol for precise delivery of unilateral trip-like perturbations during gait
Source: PLoS One. 2023 Apr 25;18(4):e0284384. doi: 10.1371/journal.pone.0284384 (PMC10128926; doi:10.1371/journal.pone.0284384)

The Treadmill-Based Tripping Perturbation

Hui-Ting Shih[1,2], Robert Gregor[3], Szu-Ping Lee[1]

[1]Department of Physical Therapy, University of Nevada Las Vegas;
[2]Baylor Scott & White Research Institute;
[3]School of Integrated Health Sciences, University of Nevada Las Vegas

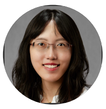

Hui-Ting Shih

OCT 22, 2022

OPEN ACCESS

DOI: dx.doi.org/10.17504/protocols.io.ewov1o8k7lr2/v1

Protocol Citation: Hui-Ting Shih, Robert Gregor, Szu-Ping Lee 2022. The Treadmill-Based Tripping Perturbation. **protocols.io** https://dx.doi.org/10.17504/protocols.io.ewov1o8k7lr2/v1

MANUSCRIPT CITATION:
Sessoms, P.H., Wyatt, M., Grabiner, M., Collins, J.-D., Kingsbury, T., Thesing, N., Kaufman, K., 2014. Method for evoking a trip-like response using a treadmill-based perturbation during locomotion. J Biomech 47, 277-280.
Zhang, F., D'Andrea, S.E., Nunnery, M.J., Kay, S.M., Huang, H., 2011. Towards design of a stumble detection system for artificial legs. IEEE Trans Neural Syst Rehabil Eng 19, 567-577.

License: This is an open access protocol distributed under the terms of the Creative Commons Attribution License, which permits unrestricted use, distribution, and reproduction in any medium, provided the original author and source are credited

ABSTRACT

The protocol utilized a side-by-side split-belt instrumented treadmill to simulate a trip-like perturbation. Compared to previously published methodologies, this new protocol is focused on delivering the perturbation at a precise timepoint during gait. Programmed treadmill belt acceleration profiles are triggered unilaterally at the instant the tripped leg bears 20-25% of the body weight in early stance phase. The belt acceleration profile starts from a comfortable walking speed, followed by a sudden slowdown, then an acceleration, and finally returning to the comfortable walking speed. The slowdown is meant to simulate the foot obstruction and the acceleration reproduces the leg-torso discoordination and the trunk forward thrust during a trip.

ATTACHMENTS

540-1125.docx 540-1124.docx 540-1126.zip

GUIDELINES

Guidelines

This protocol is designed to invoke a destabilizing condition that simulates an unexpected fall event. Using an instrumented treadmill, the protocol enables consistent and accurate delivery of postural perturbation to a test participant who is walking on the treadmill. Reliability of participants’ biomechanical responses to repeated perturbations has been established. The protocol has been further validated for distinguishing fall risk and fall recovery movement patterns between younger and older adults by our research group.

Protocol Overview

Protocol status: Working
We use this protocol and it's working

Created: Sep 30, 2022

Last Modified: Mar 31, 2023

PROTOCOL integer ID: 70693

Keywords: Fall, Trip, Stumble, Perturbation, Disturbance

System Apparatus Design

A Bertec side-by-side split-belt instrumented treadmill (Model ITC-11-20L-4, Bertec Corp., Columbus, OH, USA) has independent control of the movements of the two treadmill belts. Each belt is equipped with one force plate capturing GRF data from the walker's foot contacts. The force data are sampled at 1000Hz, time-synchronized with the VICON (Oxford Metrics, Oxfordshire, UK) motion data and streamed by the Software Development Kit (SDK) to MATLAB (MathWorks Inc., Natick, MA, USA) on a personal computer. The MATLAB program serves as the control interface to communicate between the VICON Datastream SDK and the treadmill controller. Concisely, the MATLAB program reads the vertical GRF (vGRF) from Datastream SDK, and when the pre-determined conditions are met it executes the pre-programmed perturbations via the treadmill controller. The treadmill controller receives remote control commands from MATLAB via the Transmission Control Protocol/Internet Protocol port, through which the program delivers the prescribed tripping perturbation by accelerating/decelerating the treadmill motors (Figure 1 and 2B).

Perturbation Design

The treadmill-based tripping perturbation protocol begins with establishing participants' comfortable walking speeds (CWS). During a perturbation a designated treadmill belt, either left or right, decelerates for 50ms, followed by 270ms of acceleration, and then decelerates again for 220ms to return to the CWS (Figure 2B). This profile is designed to simulate the sudden blockage of a foot followed by the momentary forward thrust of the body center of mass. Two acceleration levels are used to simulate tripping perturbations of two levels of magnitude (small vs. large) (Sessoms et al., 2014) . The acceleration magnitude utilized in the protocol is linearly scaled by the CWS. For a CWS at 1 m/s, the acceleration is either ± 6 m/s 2 (small tripping perturbation) or ± 12 m/s 2 (large tripping perturbation). This allows the delivery of more realistic magnitudes of perturbation for individuals with slower walking speeds. The magnitude of perturbation may be adjusted to the researcher's needs. The automatic triggering criteria are based on the vGRF profile with the intention to deliver the perturbation precisely during early stance phase of the tripped limb. It ensures that the tripped limb went through the full course of the velocity changes of the treadmill belt. The perturbation is triggered when the following conditions are jointly met: First, the vGRF of the tripped side has to be between 20-25% of the person's body weight. Second, vGRF that met the first condition has to be greater than the vGRF 10ms prior to ensure that the trigger would occur in the ascending phase of the vGRF typical of during the early stance phase (Figure 2A).

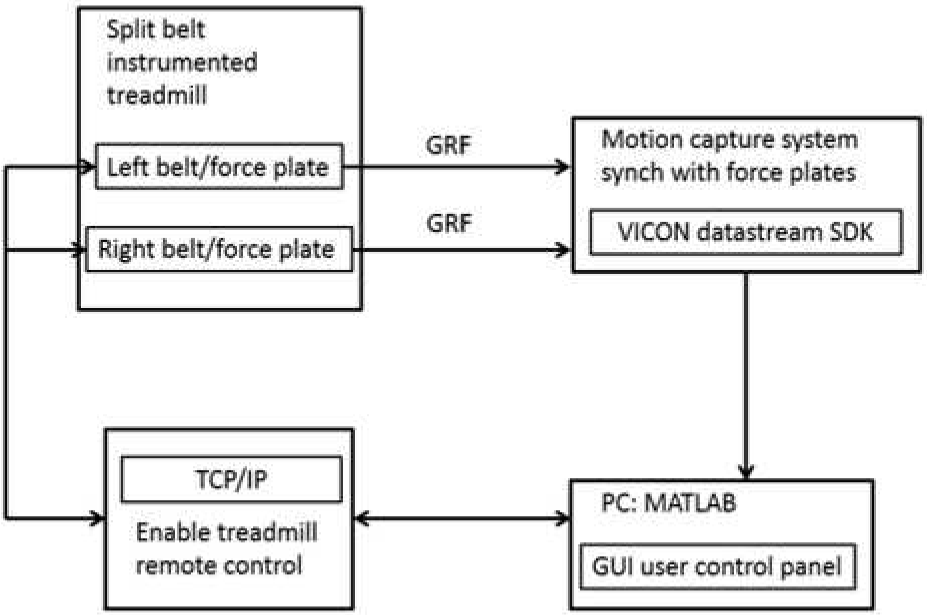

Figure 1. System apparatus. GRF: ground reaction force; SDK: software development kit; PC: personal computer; GUI: graphical user interface TCP/IP: transmission control protocol/internet protocol.

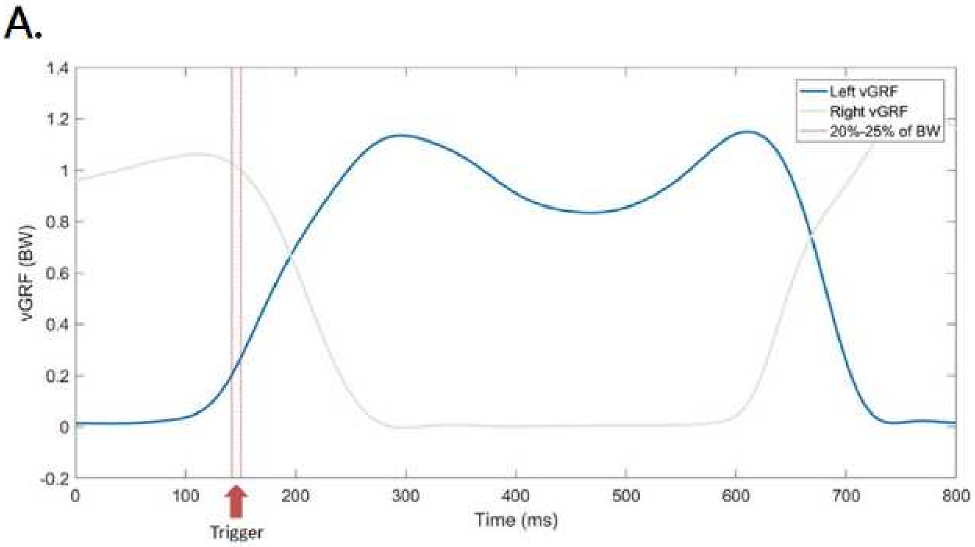

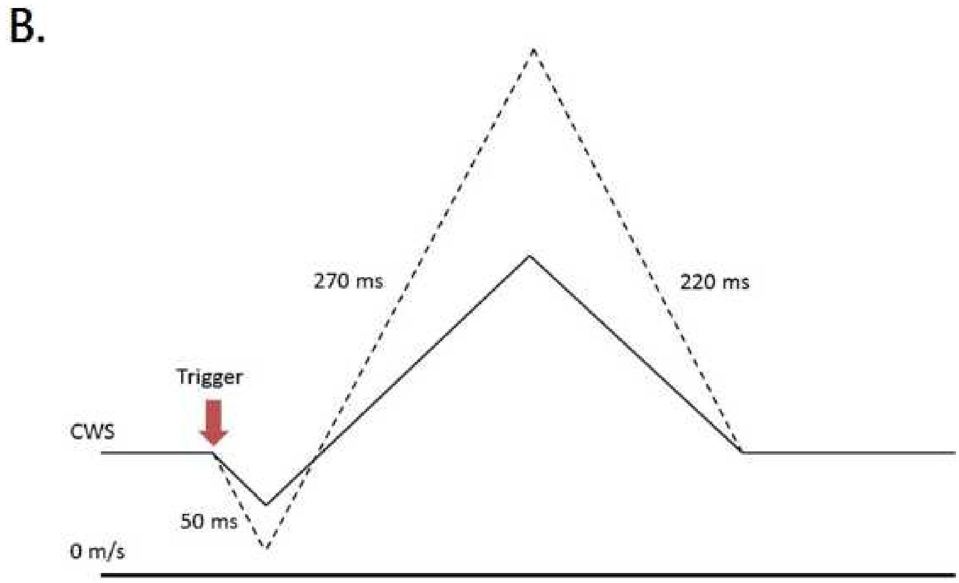

Figure 2. Perturbation Triggering Criteria and Treadmill Velocity Profile (A) GRF from both limbs and perturbation triggering criteria. Blue vGRF line is from the tripped left limb; red lines enclose the designated window of a triggering event. (B) Treadmill velocity profile. Solid line stands for a tripping perturbation with a small acceleration; dash line represents a tripping perturbation with a large acceleration.

Citations

- Sessoms, P.H., Wyatt, M., Grabiner, M., Collins, J.-D., Kingsbury, T., Thesing, N., Kaufman, K., 2014. Method for evoking a trip-like response using a treadmill-based perturbation during locomotion. J Biomech 47, 277-280.
- Zhang, F., D'Andrea, S.E., Nunnery, M.J., Kay, S.M., Huang, H., 2011. Towards design of a stumble detection system for artificial legs. IEEE Trans Neural Syst Rehabil Eng 19, 567-577.

Materials

We suggest that this protocol should only be applied to individuals who can walk independently without assistive device for at least 5 minutes. The suggestion is estimated based on the time required for familiarizing the individual to treadmill walking, fitting for the harness, and completing the protocol.

Equipment

• Bertec side-by-side split-belt instrumented treadmill (Model ITC-11-20L-4, Bertec Corp., Columbus, OH, USA)
• VICON motion capture system (Oxford Metrics, Oxfordshire, UK) with Software Development Kit (SDK)
• MATLAB version R2017b or newer

SAFETY WARNINGS

Given the protocol is triggered by a vertical ground reaction force during gait, the protocol is not applicable to individuals who are unable to walk on a treadmill. The safety of the individual should be the priority. Potential injury, pain, or discomfort may be induced by protocol in individuals with prior lower extremity orthopedic issues due to the sudden movement of the leg. Additional warning message from the treadmill manufacturer as related to the execution of the protocol is provided below: "Once remote control for the treadmill is enabled, the treadmill can execute immediate full-speed motion based on commands received from the remote source. Please ensure you have proper firewall settings in place to prevent uncommanded treadmill operation. Manual operation of treadmill controls will overwrite and disable remote control."

BEFORE START INSTRUCTIONS

Please double check the setting for fall prevention/participant protection. We strongly suggest a safety harness to be installed and properly tested before applying the protocol to any individual. To ensure participants do not hit the ground, after fitting participants to the harness, we asked them sit in the harness and put their full body weight to it like playing on the swing and bending their knees. We adjusted the tether under this circumstance so that their knees would not hit the ground even in the most severe fall event. The adjusted tether length should not interrupt the participant's gait. Padding can be added to the supporting struts and handrails to better protect the participants. In order to run the protocol, a few parameters need to be gathered including participants' comfortable walking speeds for treadmill walking in m/s (or a designated speed decided by the research team) and their body weight in kg.

The Treadmill-Based Tripping Perturbation

1 Turn on VICON motion capture system and open "Vicon Nexus".

Vicon Nexus

2 Make sure the force platforms are enabled and available.

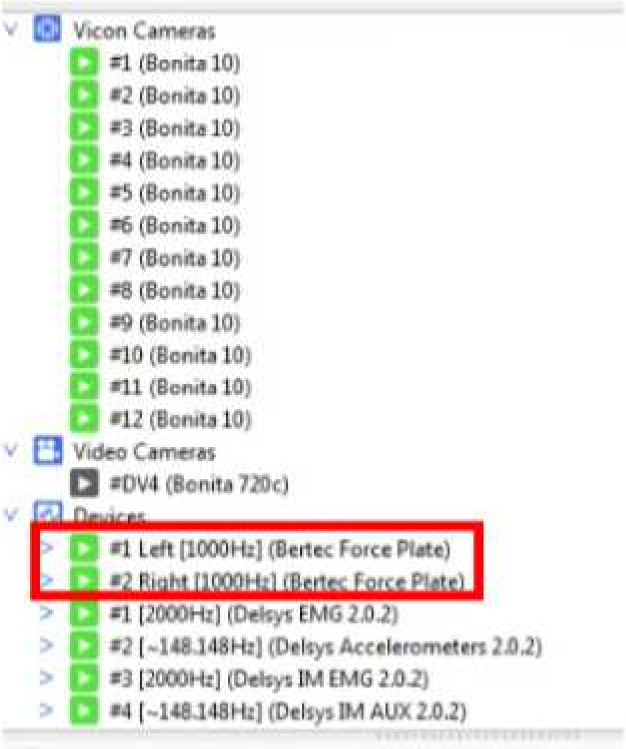

3 Turn on Bertec treadmill and open "Bertec treadmill". A treadmill control panel will pop out.

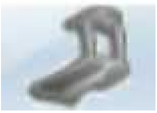

4 Click "Settings" > check "Remote TCP/IP Control".

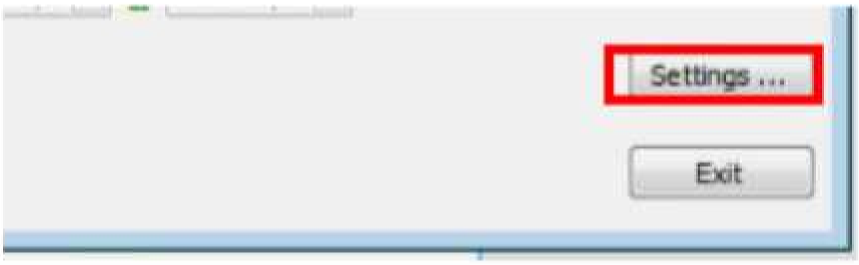

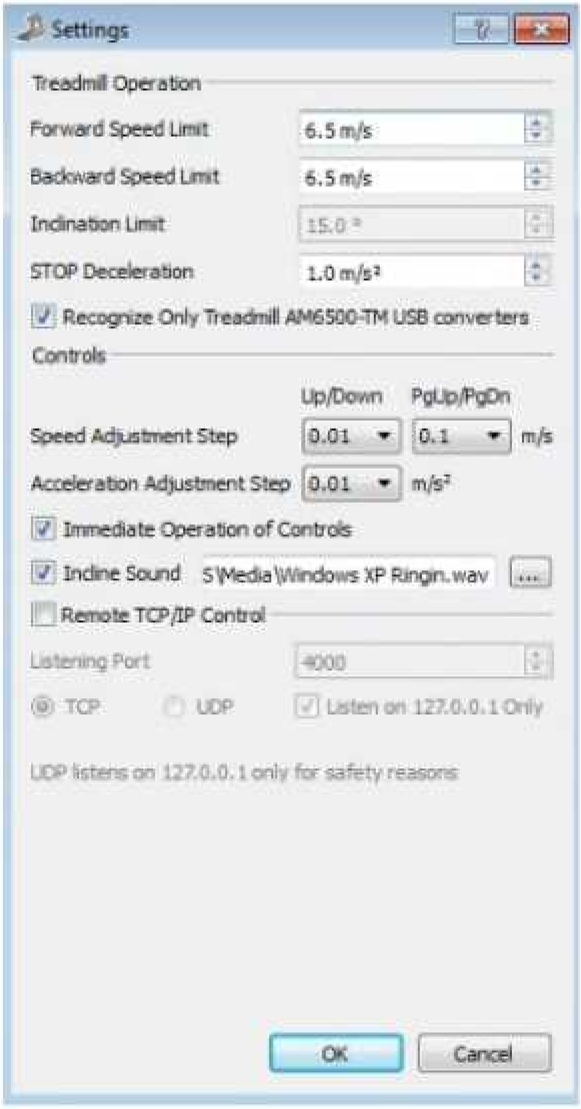

5 Click “OK” on the warning pop-out window.

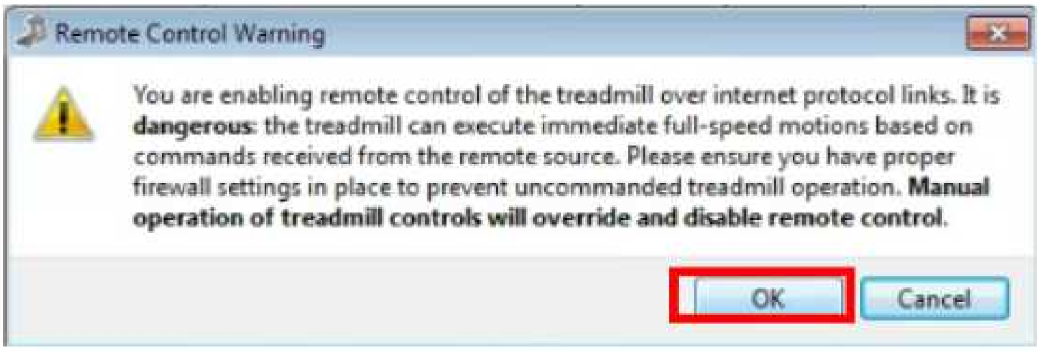

6 Click “OK” on the Settings window. All the disabled remote-control functions (grey functions in step 4) should be enabled.

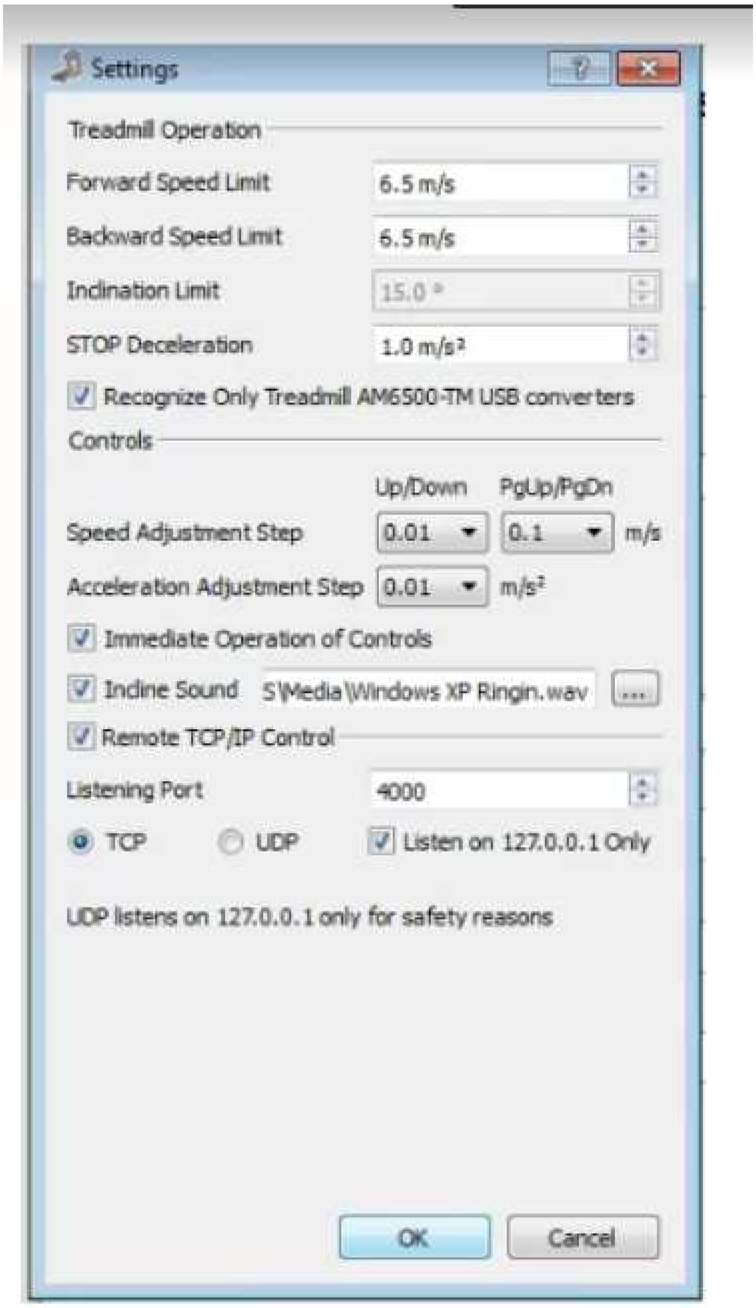

7 Click “Enable Remote Control” on the treadmill control panel.

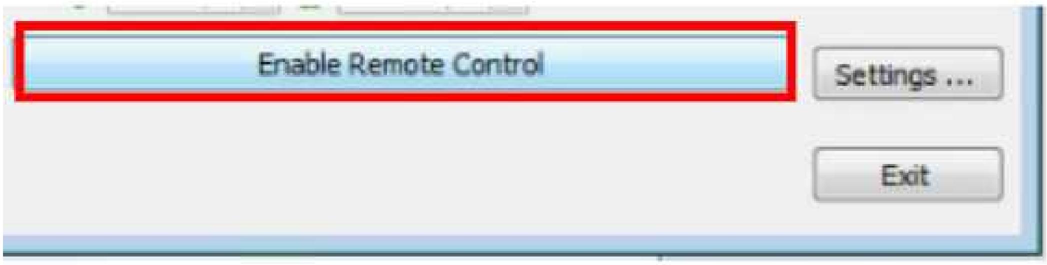

8 Open “Matlab".

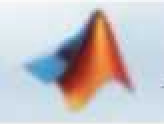

9

Open file: trip_version2.mlapp. Make sure all Matlab files are placed in the same folder.

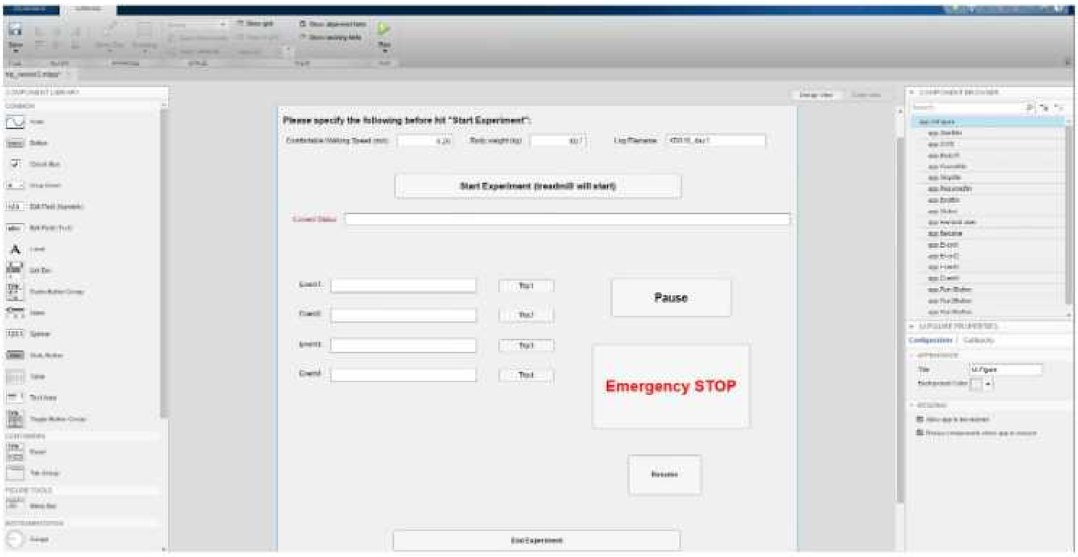

10

Run this application. An UI Figure window will pop out showing the control panel.

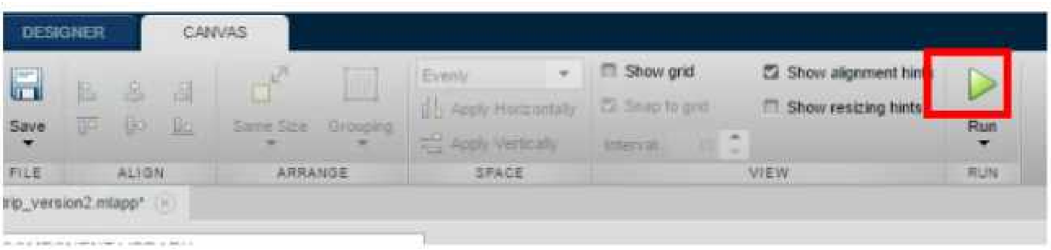

11

Input participant's comfortable walking speed (m/s), body weight (kg), and filename. After typing in the filename, "Current Status" will show "Thank you for specify filename for log".

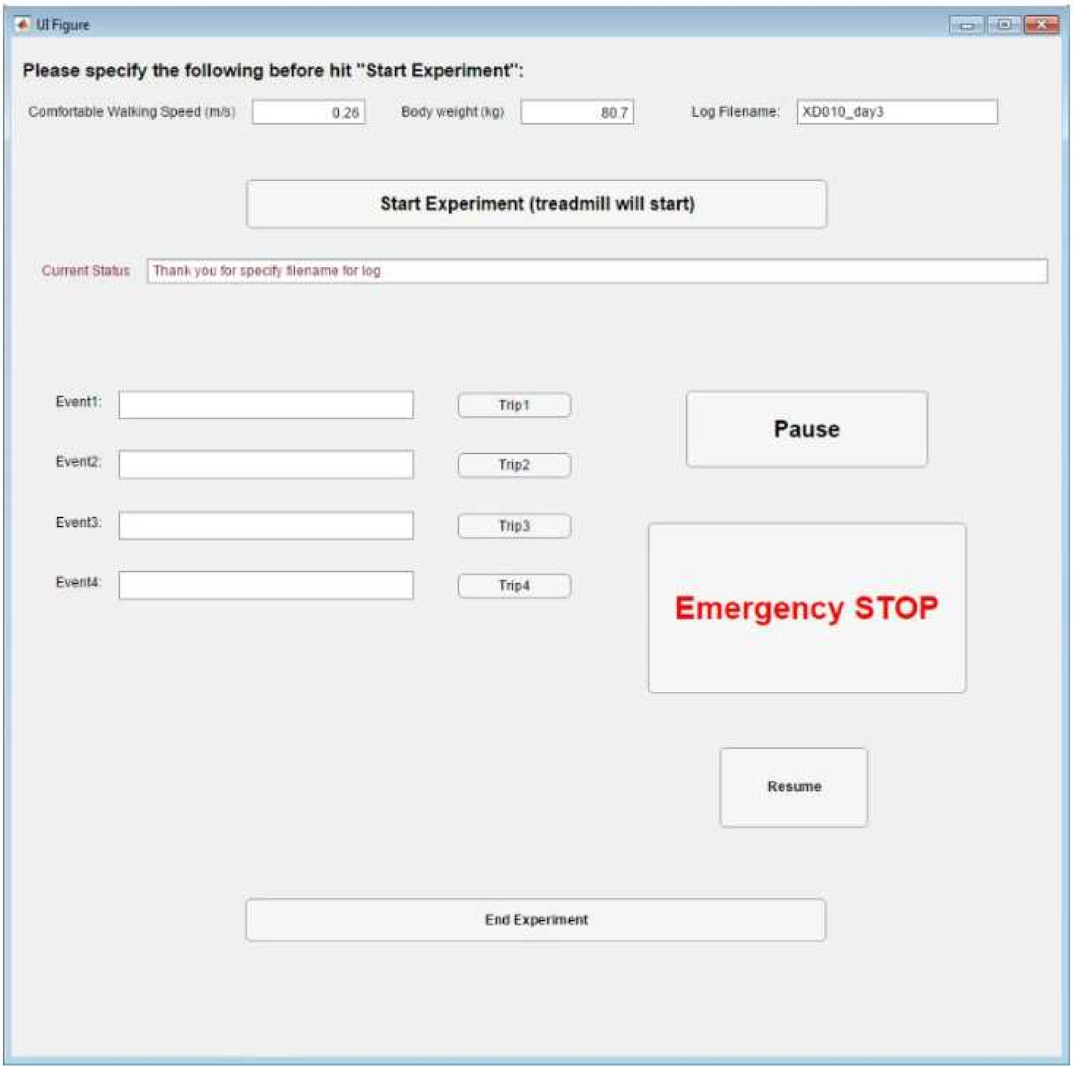

Note

The filename is for Matlab to save a log file reporting the order of perturbations delivered. More details are provided in step 16. The path for saving this log file can be modified by visiting “code view” of this app (step 9) and change line 40 (absolutepath =).

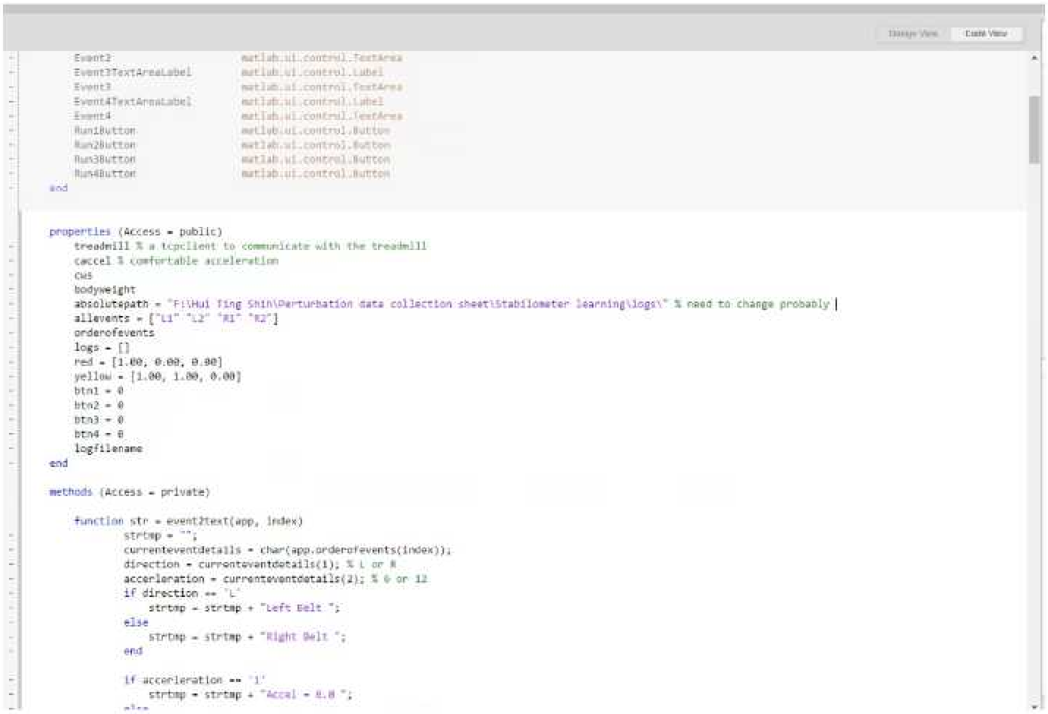

12 Click “Start Experiment”. The treadmill will start moving immediately and gradually accelerate until reaching the comfortable walking speed that was just input. “Current Status” will show “Bertec Treadmill: Connected”. In the meantime, Matlab automatically randomizes the 4 perturbation conditions (left/right x small/large) and assigns them as events 1 to 4.

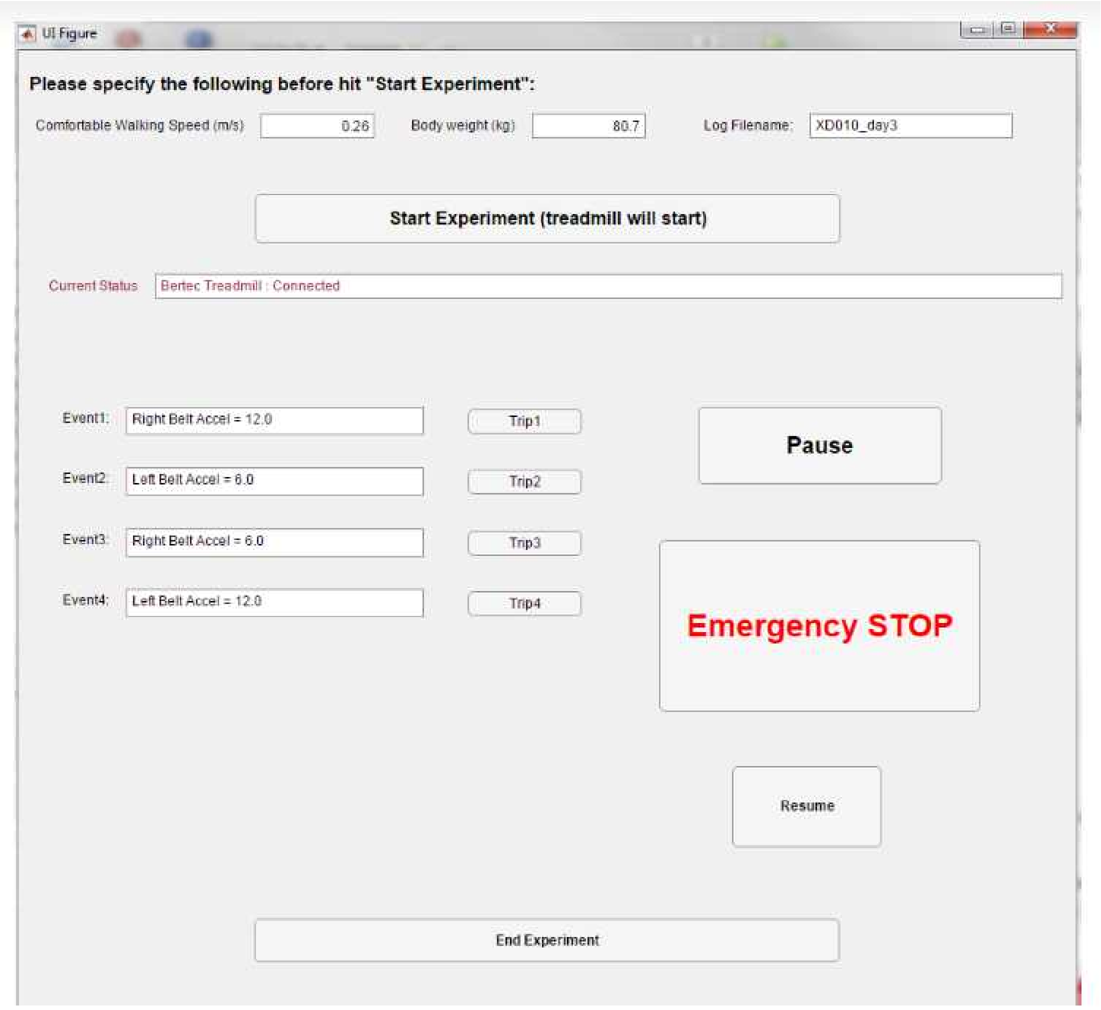

13

After participants reach a stable gait pattern, a researcher can click “Trip 1” to allow Matlab to begin screening the ground reaction force and automatically deliver the perturbation when the triggering criteria are met. Once the perturbation is delivered, the “Trip 1” button turns yellow. Matlab also shows the current status as “Waiting to Trip……” and “Trip 1 Executed”.

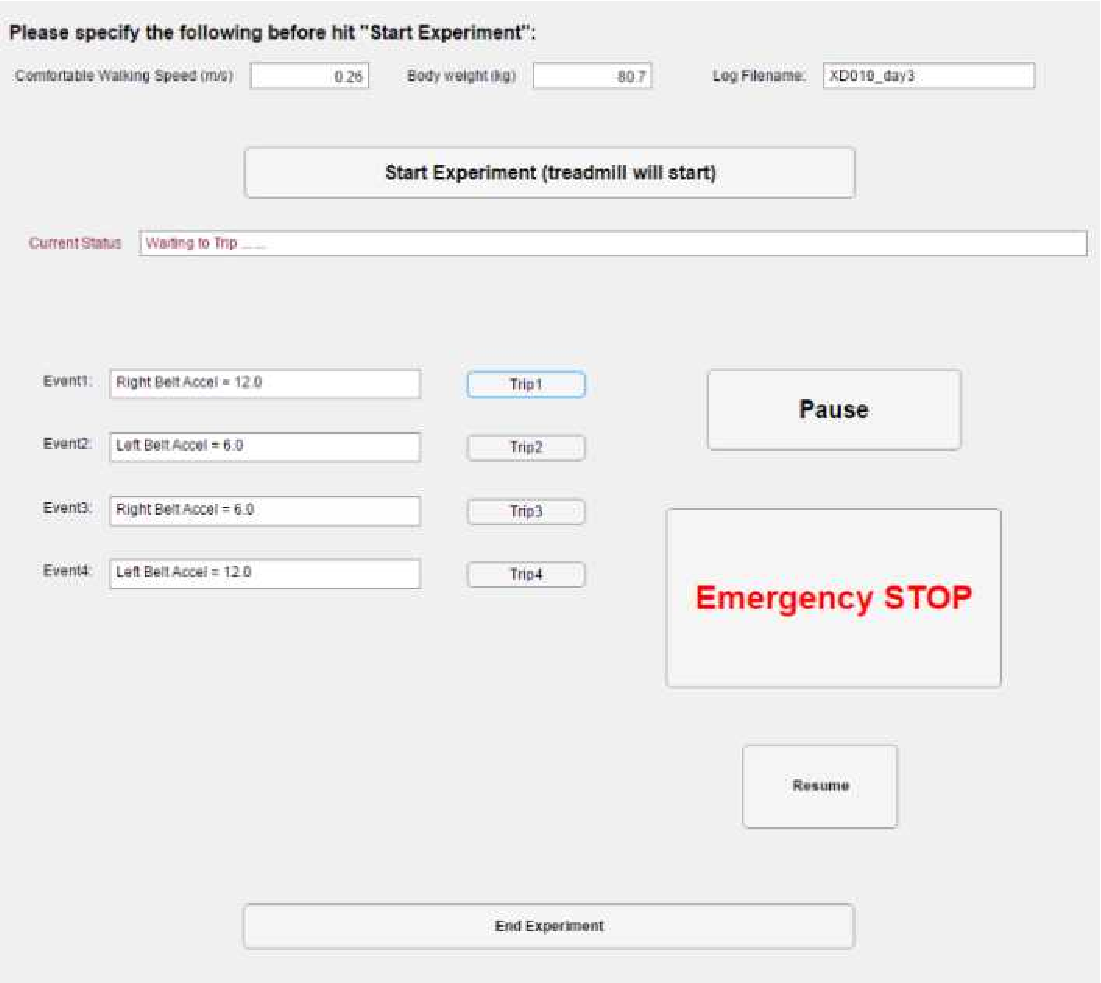

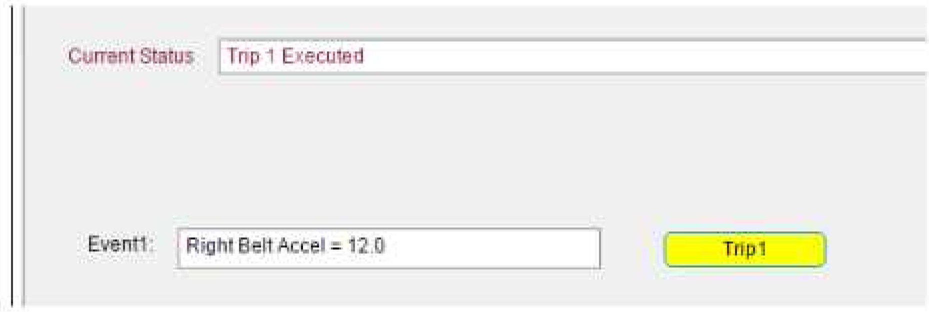

14 After a perturbation, participants may need some time to resume stable gait. Continue delivering the next perturbation by clicking “Trip 2” and repeat the same procedure for “Trip 3” and “Trip 4”.

15 The function “pause” will gradually slow down and stop the treadmill, and function “resume” will bring the treadmill velocity back and continue from where was left out. We recommend using

these functions when participants need a short break. The “Emergency STOP” will stop the treadmill right way, forcing the treadmill velocity to be zero immediately, which we recommend using it when participants completely lose balance or in an emergency. After an emergency stop, it is required to close the application and re-run it (step 10).

16 After successfully delivering all perturbations, click “End Experiment”. “Current Status” will show the complete path where the log file is saved. Matlab automatically saves a log file including the perturbation detail in sequence to the designated path with the input filename as “filename_dynamic.txt”. For example, for a trial with an input filename as “XD010_day1”, the log file will be named as “XD010_day1_dynamic.txt”. The log file includes direction (left or right), accel (acceleration; 6=small or 12=large), zForce (the vertical ground reaction value when the perturbation was delivered), and FrameNumber (the frame at which the perturbation was delivered).

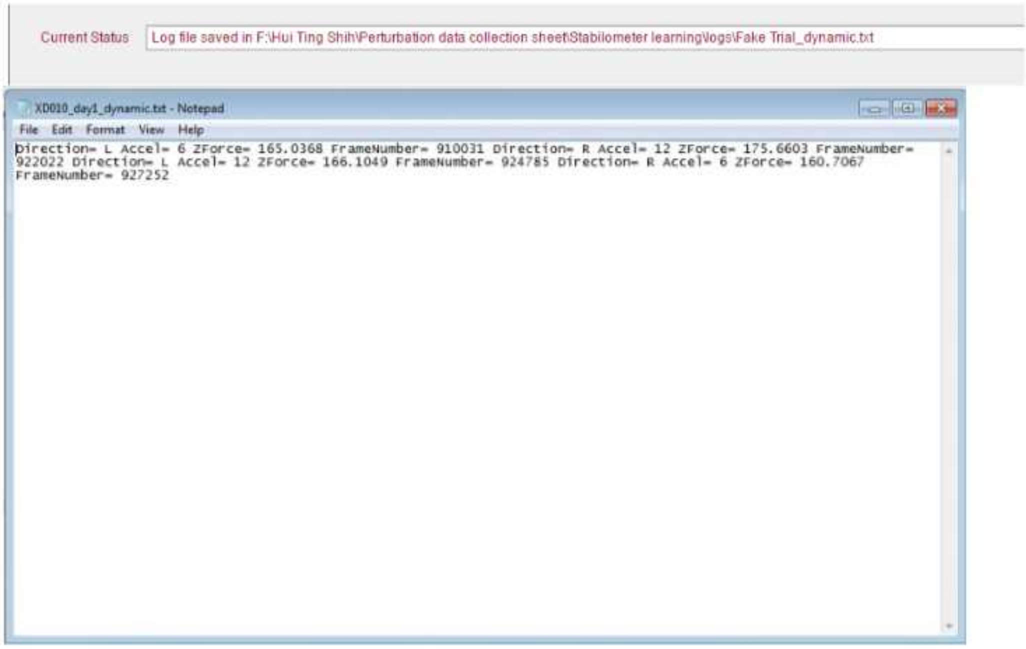

Supplement: S1 File — dx.doi.org/10.17504/protocols.io.ewov1o8k7lr2/v1. (PDF) [file pone.0284384.s001.pdf]
