# Supplementary material for: Description, reliability and utility of a ground-reaction-force triggered protocol for precise delivery of unilateral trip-like perturbations during gait
Source: PLoS One. 2023 Apr 25;18(4):e0284384. doi: 10.1371/journal.pone.0284384 (PMC10128926; doi:10.1371/journal.pone.0284384)
Supplement: Attachment — Submitted filename: Response to reviewers v2.docx [file pone.0284384.s002.docx]

Reviewer #1: Thank you for inviting me to review the article “Description, Reliability and Validity of a Ground-Reaction-Force Triggered Protocol for Precise Delivery of Unilateral Trip-Like Perturbations During Gait”.
With this Protocol Lab, the authors broaden the current knowledge on 'treadmill-based perturbation protocols', providing useful information to the research community.
The materials and methods are clear and well detailed, and the results are consistent with the purpose of the study.

RESPONSE: Thank you for taking the time to review our manuscript. We appreciate the kind words and the detail in which the review was conducted.

I have some suggestions for improving the manuscript.

• There is no justification for the sample size.

RESPONSE: We considered the test-retest reliability of our primary purpose and used a hypothesis testing approach to calculate the sample size needed (Walter et al., 1998). The hypothesis testing approach is used when the goal of the study is to show that the ICC is larger than some minimum threshold of interest, represented as ρ0 which is commonly the goal of reliability studies in exercise, sport, and rehabilitation.

For our study, we determined the minimum threshold of ICC (ρ0) as 0.7 for we hope our tool shown to have sufficiently high reliability. The anticipated ICC was set at 0.95, power at 80%, and significant level at 0.05, and the number of repeats was 2. The estimated sample size was 10.

The sample size justification has been included in P. 11-12 Line 238, 241-248.

- *Walter SD, Eliasziw M, Donner A. Sample size and optimal designs for reliability studies. Stat Med. 1998;17(1):101-110.*

• I suggest reviewing the units of measurement and reporting them as “m·s−1” instead of “m/s”

RESPONSE: We changed the units of velocity and acceleration to m·s^-1^ and m·s^-2^ respectively throughout the manuscript and table. The changes are marked in yellow.

• Where possible, highlight the significant differences also in the tables.

RESPONSE: The significant differences were denoted in the tables and marked in yellow.

• I suggest to the authors to broaden the 'Data Discussion' by also referring to what is already present in the literature.

RESPONSE: We added 5 paragraphs in the Data Discussion, which stated the strength of a treadmill-based protocol, justified the merits of precise delivery of perturbations, elucidated the value of the two acceleration profiles, and discussed the kinematic results documented from the younger and older groups.

Reviewer #2: I want to thank the Editors and the Authors for the chance to review this manuscript.
This study aims to develop and validate a treadmill-based protocol for generating unanticipated perturbations during walking.

The methodology is interesting and well-reported. However, the background and the discussion of the findings lack some details, potentially leaving the reader without compelling insights regarding the practical applicability of the study outcomes. I would suggest substantially improving and expanding these sections to increase the manuscript value.

In addition, as a practical note, I must report that the manuscript does not include line numbering, as suggested in the journal guidelines, making the review process more difficult.

RESPONSE: Thank you for your time and effort spent on reviewing our manuscript. The suggestions helped us improve our manuscript substantially.

We inserted continuous line numbers in the revision.


The detailed comments are listed below:

Page 4: “Researchers have focused on trunk kinematics…”, please elaborate more on the past findings to provide a more comprehensive background.

RESPONSE: We elaborated on the past literature and findings regarding the trunk kinematics on P.4 Line 67-73, justifying the selection of outcome measures, namely peak trunk flexion angle, in the current study.

Pavol et al. and Owing et al. examined 39 kinematic variables during tripping in healthy older adults. They found that peak trunk flexion angle was not only related to but also a significant indicator of the likelihood of falls. As mounting evidence accumulated, Grabiner and Kaufman reviewed fall-related studies and identified peak trunk flexion angle as an appropriate risk biomarker for falls. A recent study further showed that trunk control is a critical and task-specific parameter in avoiding falls after perturbation.

Pages 4-5: what does “the precision of perturbation delivery” affect? The kinematics or the reaction response? Please describe in more detail the previous studies limitations that create a gap in the literature that the present study aims to fill.

RESPONSE: The onset timing of the tripping event largely determines what strategy will be selected (Eng et al., 1994). Delivering the perturbations at precise timing is critical because the consequent recovery responses, both reaction response and kinematics, are different. Three different categories of tripping fall recovery strategies have been identified in healthy persons.

• Elevating Strategy: When performing an elevating strategy, the tripped limb is typically flexed to prepare for a recovery step while the contralateral (non-tripped) limb support and elevate the body center of mass. It is the predominant strategy if the trip occurs during early swing phase of the tripped limb.

• Lowering Strategy: During lowering strategy, the tripped limb is quickly lowered to the ground immediate after the obstruction. The tripped limb serves as the support limb during the perturbation to enable the recovery step from the contralateral, non-tripped limb. It is usually a response to a trip occurring during late swing phase of the tripped limb.

• Reaching Strategy: If the trip occurs during late swing phase, an individual may alternatively use a reaching strategy, prolonging or maximizing the flexion of the tripped limb, especially the hip, to execute an extra big forward step to go over the obstacle. In this case, the contralateral, non-tripped limb is the support limb.

We briefly elaborated the causal relationship between onset timing and recovery strategy selection on P.5 Line 88-91 to explain why precise timing of perturbation delivery is important for this field. To clarify, we reworded Line 85 as “the precise timing of perturbation delivery”.

- *Eng, Janice J., David A. Winter, and Aftab E. Patla. "Strategies for recovery from a trip in early and late swing during human walking." Experimental brain research 102 (1994): 339-349.*

Page 5: the purpose “to further improve biomechanical evaluations...” seems way too broad. Please, be more specific in formulating aims and hypotheses for the study.

RESPONSE: We modified the purpose statement (P. 6 Line 102-105) which now reads

The purpose of this study was to develop a GRF-triggered treadmill perturbation method that emphasizes precise and reproducible timing control of the tripping perturbation delivery. Specifically, we proposed a protocol that was designed to repeatedly deliver tripping perturbations during early stance phase by referencing to GRF.

Page 7: how was CWS assessed and set for each subject? Please explain.

RESPONSE: We included details regarding how the CWS was established on P. 7 Line 139-143.

Participants were asked to self-select a walking speed during a 1-minute treadmill walking trial. The speed was finetuned by participant’s request or adjusted if the participant did not walk in the middle of the treadmill. We increased the speed if the participant stepped to the front edge of the treadmill and vice versa, and they must be able to stay with the speed for 1 minute.

Page 7: what does “more realistic magnitudes” mean in this context? It seems vague terminology.

RESPONSE: We eliminated the vague terminology and revised the sentence on P.8 Line 151-153.

As a constant acceleration would create a more challenging perturbation for individuals with a slower walking speed, this scale allowed the protocol to accommodate individuals with various walking speeds.

Page 8: why the delay was quantified? Please explain.

RESPONSE: As Figure 1 shows, in order to run the protocol, multiple systems must coordinate and communicate with one another. Even though we programmed MATLAB to deliver the perturbation when the vGRF was about 20% of the person’s body weight, we did not know if the actual perturbation occurred at the wanted phase because there might be a communication delay in the system. For example, when the force plate detected the GRF of 20% body weight, it took time for VICON datastream SDK to capture the signals and send them to MATLAB. The other way around, when MATLAB sent out the command to deliver the perturbation, it took time for the treadmill motor to receive and execute the command. That’s been said, given the communication of multiple systems, it was likely that when the treadmill belt velocity really changed, it was no longer the instance of vGRF being 20% body weight. If the delay was too long, the perturbation might occur in a later phase of gait rather than what we set (early stance phase), and that was the reason we quantified the delay as well as confirmed the phase of gait when the belt velocity started changing.

Page 8: I doubt whether studying kinematics differences between the two age groups may be considered a “validity” analysis. Demonstrating that there are differences would not mean that the protocol is valid per se. I would reword this particular purpose.

RESPONSE: We appreciate the suggestion and reworded “validity”. “Utility” was used instead throughout the manuscript.

Page 9: How long the walking trials were? How many trials did each subject perform, and how were the kinematic data averaged and extracted?
Please provide more details.

RESPONSE: We added the details below to P. 10 Line 204-210. The durations of the walking trials varied person by person, generally less than 5 minutes. A few situations could make the duration longer. First, participants requested breaks between perturbations. Second, when a person fell and could not return to walk, we stopped the treadmill, repositioned the participant, and completed the rest of the trials. Third, after a perturbation, we ensured by observation that they walked steadily again before delivering the next perturbation, and the time each person needed to resume a steady gait was different.

Each visit, each participant performed 4 perturbations: small perturbations to the left limb and to the right limb, large perturbations to the left limb and to the right limb. The information is provided on P. 10 Line 201-203. We do not reiterate perturbations with the intention to minimize the learning effect of repeated exposure in the same day.

Three events, perturbation onset, contralateral limb contact, and the tripped limb contact were chronologically identified in reference to foot position and vGRF magnitude. Peak trunk flexion angle, obtained after perturbation onset and before tripped limb contact, was extracted. Each participant’s left and right tripping trials at the same acceleration were averaged and used for analysis. The information is provided on P. 11 Line 229-234.

Page 12: how was the trunk kinematics “consistency” between the visits assessed? Please explain.

RESPONSE: Since “consistency” is a vague and inaccurate term in regard of the purpose of this analysis, we rephrased the sentence as “Peak trunk flexion angles during both the small and large perturbations were reliability elicited between the visits.”

Page 12: usually, older adults have a significantly lower preferred walking speed than younger. However, this study reports no differences. What do the Authors think about this result, and how can they explain it?

RESPONSE: The older group in our study was not elderlies but most of them were in their 50s (frequency table attached below). Moreover, they were asymptomatic adults. A previous study has shown that in asymptomatic adults, the preferred walking speed significantly slowed down after they were >70 years old (Novaes et al., 2011). Collectively, we think the relatively young age in our older group resulted in the similar CWS in the two groups. To avoid confusion, we renamed this group the “middle-aged” group.

|  | | Frequency |
| --- | --- | --- |
| Age | 51 | 2 |
|  | 54 | 1 |
|  | 56 | 1 |
|  | 57 | 2 |
|  | 59 | 2 |
|  | 60 | 1 |
|  | 67 | 1 |
|  | Total | 10 |

- *Novaes, Rômulo D., Aline S. Miranda, and Victor Z. Dourado. "Usual gait speed assessment in middle-aged and elderly Brazilian subjects." Brazilian Journal of Physical Therapy 15 (2011): 117-122.*

Page 13: the discussion is too short of discussing the interesting results effectively. Please elaborate more, providing a comparison to previously published results and practical applications.

RESPONSE: We added 5 paragraphs in the Data Discussion, which stated the strength of a treadmill-based protocol, justified the merits of precise delivery of perturbations, elucidated the value of the two acceleration profiles, and discussed the kinematic results documented from the younger and older groups.

The quality of the figures is low. They should be improved.

RESPONSE: We increased the resolution of all figures and hope the quality is improved.
